# A Biopolymer System Based on Chitosan and an Anisotropic Network of Nickel Fibers in the Hydrogen Evolution Reaction

**DOI:** 10.3390/molecules31010150

**Published:** 2026-01-01

**Authors:** Guliya R. Nizameeva, Elgina M. Lebedeva, Viktoria V. Vorobieva, Evgeniy A. Soloviev, Ruslan M. Sarimov, Irek R. Nizameev

**Affiliations:** 1 Arbuzov Institute of Organic and Physical Chemistry, FRC Kazan Scientific Center, Russian Academy of Sciences, Kazan 420088, Russia; guliya.riv@gmail.com (G.R.N.);; 2 Department of Physics, Kazan National Research Technological University, Kazan 420015, Russia; 3 Prokhorov General Physics Institute of the Russian Academy of Sciences, Moscow 119991, Russia; 4 Department of Nanotechnology in Electronics, Kazan National Research Technical University Named After A.N. Tupolev-KAI, Kazan 420111, Russia

**Keywords:** biopolymer matrix, chitosan, anisotropic network of nickel fibers, hydrogen evolution reaction, electrocatalytic activity

## Abstract

In this study, we developed a method for creating an active layer based on a composite material consisting of chitosan and an anisotropic network of nickel fibers (***Chitosan/Ni + NiFs***). Using this chitosan biopolymer matrix and anisotropic network, we achieved a high specific surface area for the catalytic material, high lateral conductivity for the layer, and stable characteristics, ultimately leading to increased overall electrocatalytic activity in the hydrogen evolution reaction (HER). Through linear voltammetry and impedance spectroscopy, we identified the mechanism and kinetics of the HER in the developed system. The overpotential of the electrochemical reaction was 213 mV at a current density of 10 mA/cm^2^. Chromatographic analysis confirmed the effectiveness of the ***Chitosan/Ni + NiFs*** system in the HER. Our results show how the chitosan biopolymer matrix and oriented nickel fiber network influence charge transfer and electrode reactions, as reflected in the activation energies of hydrogen bonds on the electrocatalytic layers. These findings show that it is feasible to combine a biopolymer matrix and an anisotropic nickel fiber network to create effective electrocatalysts. This approach enables the development of environmentally friendly electrolytic hydrogen production technologies.

## 1. Introduction

The development of effective electrocatalysts for the hydrogen evolution reaction (HER) is urgently needed due to the global energy crisis. The global economy remains critically dependent on fossil fuels, which creates systemic environmental problems. With energy consumption expected to increase and hydrocarbon reserves depleting, the development of renewable energy sources is becoming a key area of sustainable development [1,2,3,4]. Hydrogen energy plays a key role in this process. Hydrogen offers several advantages as an energy carrier: a high energy density, zero carbon footprint, and a virtually unlimited raw material base. Although modern electrolytic hydrogen production is inferior in its cost efficiency to steam methane reforming, water electrolysis offers a path to a carbon-neutral cycle when integrated with renewable energy sources (solar, wind, and hydroelectric power plants). Despite significant progress in electrocatalysis, economic factors and the limited availability of catalytic materials remain key factors limiting the large-scale implementation of water electrolysis technology. The most effective hydrogen evolution reaction catalysts are traditionally based on noble metals (Pt, Ir, Ru) [5,6,7] or rare-earth metal alloys [8], which significantly increases the cost of the systems and hinders their commercialization. In addition to cost, there is the problem of limited natural reserves of these metals, which creates additional risks for the sustainable development of hydrogen energy. Therefore, a pressing scientific challenge is the development of highly efficient and stable electrocatalytic materials for the HER based on readily available and abundant elements.

The criteria for developing efficient electrocatalysts for the hydrogen evolution reaction involve three fundamental parameters: the intrinsic catalytic activity of the material, a developed specific surface area providing a large number of active sites, and long-term stability under operating conditions. These characteristics directly determine the magnitude of the electrochemical reaction overpotential required to achieve high current densities. Consequently, the development of highly efficient HER catalysts is based either on increasing the specific surface area by creating structures with the highest possible surface area [8,9,10,11,12] or on synthesizing materials with high intrinsic activity [13,14,15,16,17,18]. Both approaches aim to minimize the overpotential of the HER and increase the energy efficiency of water electrolysis. Modern approaches to increasing the specific surface area of catalysts primarily involve the formation of three-dimensional network structures with high porosity [19,20,21,22], which creates a developed surface area and increases the area accessible to reactants. Another promising direction area is the design of hierarchical structures combining various levels of porosity—micropores, mesopores, and macropores [23,24,25,26]—which optimizes mass transfer processes during electrochemical reactions. The use of nanostructured catalysts, such as needle-shaped nanoparticles and nanoflowers with a high area-to-volume ratio [27,28,29,30,31], also leads to an increase in specific surface area. A further increase in the active area of catalysts can be achieved through controlled synthesis and surface modification methods, such as electron beam processing [32,33] and vacuum deposition. These methods allow for the targeted formation of functional groups, control of the degree of crystallinity, and control of the chemical composition of surface layers. Particular attention should be given to anisotropic metal fiber networks—three-dimensional structures in which the metal fibers are oriented predominantly in specific directions, creating unique anisotropic properties. These networks provide a high specific surface area, improved electrical conductivity, and optimal electrical charge distribution, which reduces localized overheating and increases catalyst efficiency [34,35,36,37]. The high porosity and directional structure of these metal networks improve mass transfer and the accessibility of active sites, while also increasing the catalyst’s resistance to mechanical deformation.

Modification of the electrode surface with biopolymers is another promising direction for increasing the specific surface area of catalysts. Most natural biopolymers are chain molecules with repeating units [38], such as polysaccharides [39] (pectin, cellulose, chitosan, chitin, alginate), proteins [40] (gelatin, silk), and others. Due to their excellent biocompatibility and biodegradability, biopolymers are an attractive “green” alternative to traditional polymers [41]. These materials, having a natural porous structure and a rich functional composition, are capable of forming bulk and highly porous coatings on the electrode surface, which allows them to be used as a three-dimensional matrix for the deposition of catalytic materials. The use of a three-dimensional biopolymer carrier improves the distribution of active components and ensures stable retention of catalytic nanostructures on the electrode surface due to improved adhesion. In addition, modifying electrodes with a biopolymer matrix improves their electrical conductivity [42,43,44]. Examples of successful research include the development of electrodes modified with biopolymer coatings, on the surface of which metal nanoparticles are deposited [45]. For example, in [46], a comprehensive study was conducted on the synthesis and characteristics of a hybrid electrocatalyst based on a chitosan matrix (Chi), polypyrrole (PPy), and gold nanoparticles for the hydrogen evolution reaction. The authors used chitosan as a biopolymer base, where its unique film-forming properties ensured a uniform distribution of gold nanoparticles in the PPy-Chi composite system. Electrochemical measurements demonstrated a significant effect of the system’s composition on the kinetic parameters of the HER. The original electrode with a polypyrrole (PPy) coating was characterized by a high Tafel slope (234 mV/dec), whereas the introduction of chitosan into the polymer matrix reduced this value to 192 mV/dec. The authors explain the observed effect as being due to the intercalation of chitosan into the polypyrrole chains, which led to an improvement in the electronic conductivity of the system. Further modification of the coating with gold nanoparticles (2.09 wt%) allowed for a further decrease in the Tafel slope to 152 mV/dec. Impedance studies confirmed a decrease in charge transfer resistance from 2.1 kOhm for PPy to 0.9 kOhm for PPy-Chi. The deposition of gold nanoparticles on the PPy-Chi composite substrate resulted in a decrease in charge transfer resistance to 0.6 kOhm. Thus, the authors concluded that the combined effect of three factors—an increase in the active surface area due to the biopolymer matrix, an increase in electrical conductivity due to the introduction of gold nanoparticles, and the proton conductivity of chitosan, which ensures the delivery of H^+^ ions to the catalytic sites—led to a significant increase in the efficiency of the electrocatalytic hydrogen evolution process. Another study [47] examined SiC-based photocatalytic systems with biopolymer matrices. Biopolymers (cellulose, chitin, chitosan, sodium alginate) acted as structure-forming templates on the SiC surface and as charge transfer mediators. In the course of the study, biopolymer-SiC/MoS_x_ and biopolymer-SiC/Pt hybrid materials were obtained via the photodeposition of MoS_x_ and Pt nanoparticles from aqueous solutions of (NH_4_)_2_MoS_4_ and H_2_PtCl_6_∙6H_2_O, respectively, on the surface of biopolymer-SiC composite. In the photocatalytic hydrogen evolution reaction, the sodium alginate-SiC/MoS_x_ composite demonstrated the best photocatalytic activity, exceeding the performance of pure SiC by 158 times, that of pure sodium alginate by 34 times, and that of the sodium alginate-SiC binary system by 13 times. The efficiency of solar energy conversion into hydrogen for this catalyst reached 19.83%. The authors claimed that the use of a biopolymer matrix prevents the aggregation of MoS_x_ and Pt nanoparticles during deposition and leads to a uniform distribution of these particles on the composite surface. This promotes more efficient separation of photoinduced charge carriers, making the developed material promising for use in sustainable hydrogen energy. In [48], the authors used a PANI-chitosan composite film to modify a graphite electrode. This film was then used as a biopolymer matrix for the deposition of platinum Pt nanoparticles. It turned out that the use of a chitosan polymer matrix led to a uniform distribution of Pt nanoparticles on the PANI-chitosan surface. In the hydrogen evolution reaction for the PANI-chitosan/Pt system, the current density increased by 12 times compared to that for PANI-chitosan, and the onset potential of the reaction shifted positively to 0.5 V.

Thus, the above examples demonstrate that modifying the electrode surface with biopolymers significantly increases the specific surface area of the catalysts and their efficiency in the hydrogen evolution reaction [49]. It is worth noting that the use of biopolymers to modify the electrode surface in the hydrogen evolution reaction is also important from an environmental perspective [50]. Biopolymers are natural, biocompatible, and biodegradable materials with reduced environmental impact compared to synthetic polymers [51,52]. Hydrogen energy, aimed at sustainable development, requires components that not only increase equipment efficiency but also minimize environmental impact [53,54]. Biopolymer coatings promote stable and environmentally friendly operation of electrodes [55], as they emit no toxic products during operation and decompose in natural conditions, thereby contributing to the development of more environmentally friendly, sustainable, and safe hydrogen production technologies.

In this work, a technique combining two complementary approaches is used to increase the specific surface area of electrocatalysts: the creation of an oriented network of metallic nickel fibers (***NiFs***) and the use of a chitosan-based biopolymer matrix as a support for ***Ni*** and ***Ni + NiFs*** layers. The first approach involves forming a network of nickel fibers with controlled orientation, which provides a developed surface and efficient charge transfer pathways. The second approach involves the use of a chitosan matrix, which creates additional roughness due to its unique film-forming properties and ensures stable retention of the ***Ni*** and ***Ni + NiFs*** layers on the electrode surface. In this investigation, we examine the potential of a chitosan framework for the controlled electrochemical deposition of metallic nickel and nickel fibers. This study focuses on the influence of the biopolymer matrix and the network of oriented nickel fibers on the surface morphology and electrocatalytic activity of the synthesized coatings in the hydrogen evolution reaction. Although combinations of chitosan with metal fillers are indeed known, the use of anisotropic ***Ni*** fibers is a key difference, providing novelty in several ways. Anisotropic fibers create a low-threshold conductive 3D network, which is impossible with isotropic particles. This results in high electrical conductivity with an exceptionally low metal content. To our knowledge, this is the first study to use a chitosan + oriented Ni network composite as an electrocatalyst in the hydrogen evolution reaction. Anisotropic coatings, like nonequilibrium materials in general, exhibit significant differences from isotropic structures with the same chemical composition.

## 2. Results and Discussion

### 2.1. Morphology of Chitosan, Chitosan/Ni, and Chitosan/Ni + NiFs

**Figure 1:**
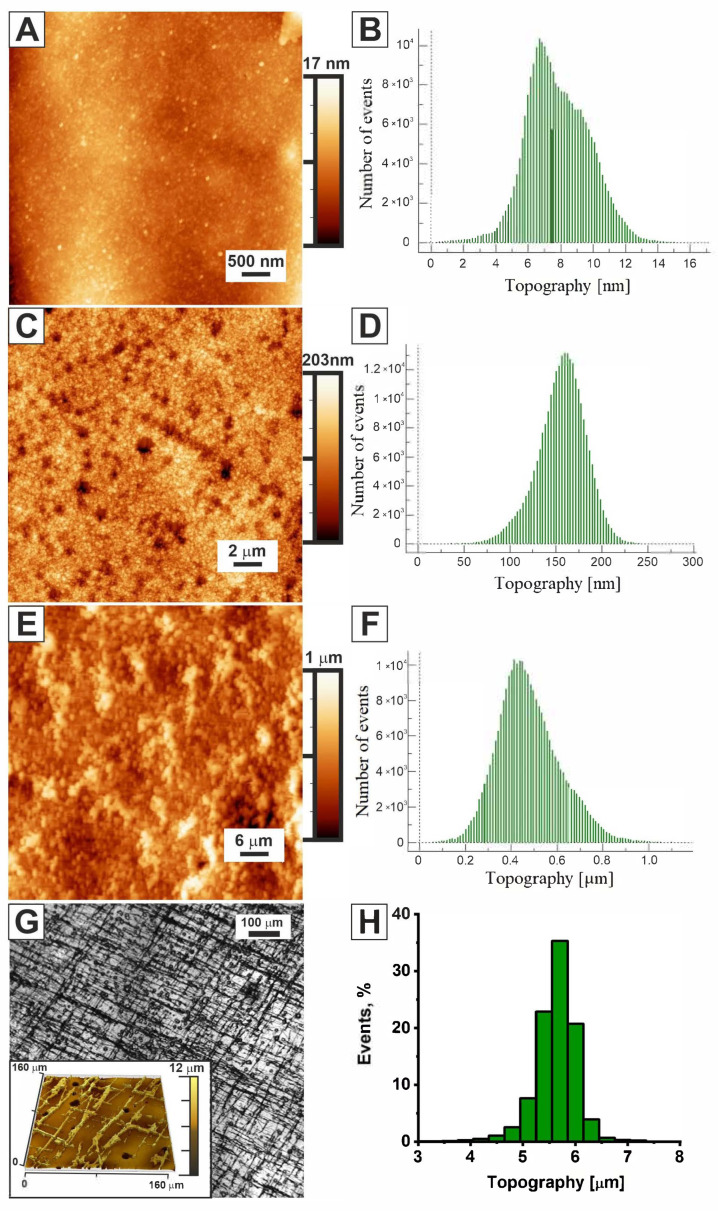
AFM images of the surface morphology of chitosan deposited on the surface of a glassy carbon (GC) electrode (***Chitosan***) (**A**), nickel electrodeposited for 90 s on the surface of a GC electrode (***Ni***) (**C**), and nickel electrodeposited for 90 s on a layer of chitosan pre-deposited on the surface of a GC electrode (***Chitosan/Ni***) (**E**), and the topographic histograms corresponding to the AFM images (**B**,**D**,**F**,**H**); an optical image of the oriented network of nickel fibers deposited on ***Chitosan/Ni*** (***Chitosan/Ni + NiFs***) (**G**) (the inset is a surface image obtained via 3D confocal microscopy).

The aim of this study was to create composite electrocatalytic materials using a chitosan-based biopolymer matrix. In this work, we explored the potential of a chitosan framework for the controlled electrochemical deposition of metallic nickel and nickel fibers. The primary focus was on the influence of the biopolymer matrix on the surface morphology and electrocatalytic activity of the synthesized coatings in the hydrogen evolution reaction. Chitosan film has low intrinsic conductivity. However, our strategy was to use it as a functional composite matrix, not a standalone material. Chitosan was used as a functional platform for the highly dispersed inclusion of catalyst ***Ni*** nanoparticles, which increases the availability of active sites. The functional groups of chitosan in the acidic environment of the electrolyte promoted the transfer of protons to active centers. The resulting composite exhibited satisfactory overall conductivity. The surface morphology of the chitosan film deposited on a glassy carbon electrode was investigated using atomic force microscopy (AFM) (Figure 1A). The AFM image shows the surface of the chitosan film (***Chitosan***) consisting of fine-grained nanostructures uniformly distributed over the entire surface. These nanostructures range in size from 40 to 120 nm, and the average grain size is approximately 70 nm. According to the topographic histogram (Figure 1B), the height difference over a 5 × 5 μm^2^ area ranges from 0 to 15 nm, and the surface roughness is 2.2 ± 0.3 nm. Figure 1E shows an AFM image of the catalyst layer obtained via the electrodeposition of nickel on a pre-applied chitosan layer (***Chitosan/Ni***), and the corresponding AFM topographic histogram is displayed in Figure 1F. Also shown for comparison is an image of the surface morphology of a nickel (***Ni***) layer electrodeposited on the surface of a glassy carbon electrode in the absence of a chitosan layer (Figure 1C). A comparative analysis of their morphology demonstrates a significant difference in surface structure between the two types of coatings. It is evident that in the absence of chitosan, the electrodeposited ***Ni*** forms a two-dimensional film consisting of compactly arranged spherical nanoparticles with sizes ranging from 50 nm to 150 nm. The surface roughness is 25 ± 4 nm. In the presence of a pre-applied chitosan layer, as expected, nickel forms a layer with a developed surface and complex morphology.

Filamentous structures and island-like nickel agglomerates are clearly visible on the surface of the chitosan matrix. According to the topographic histogram (Figure 1F), the height difference over a 60 × 60 µm^2^ area ranges from 100 to 1000 µm, further confirming the presence of protruding nickel formations on the surface of the chitosan matrix. The length of the filamentous structures reaches 15 µm, and their width is 4.5 µm. The formation of a highly developed surface is also confirmed by the high roughness value, which, for the ***Chitosan/Ni*** layer, is 125 ± 20 nm. This structure is promising for use as an electrocatalytic layer, as it combines the conductive properties of nickel with a high specific surface area provided by both the biopolymer matrix and the nickel structures themselves.

**Figure 2:**
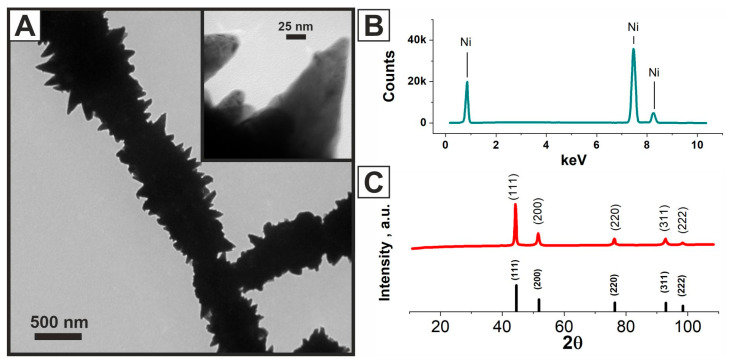
TEM image of ***NiFs*** (**A**), EDX spectrum of ***Chitosan/Ni + NiFs*** (**B**), and XRD pattern (**C**).

Since the AFM methodology has limitations related to surface roughness, the oriented nickel fiber network deposited on the ***Chitosan/Ni*** layer (***Chitosan/Ni + NiFs***) was examined using an optical microscope and 3D confocal microscopy. Figure 1G is an optical image of the ***Chitosan/Ni + NiFs*** layer, showing that the nickel fibers on the surface of the ***Chitosan/Ni*** layer are arranged in a highly oriented network. The highly developed surface structure of nickel fibers is clearly visible in TEM images (Figure 2). The use of an oriented nickel fiber network is expected to provide a high specific surface area, since the three-dimensional network structure creates a larger contact area with the reactants, as compared to continuous materials or random particle aggregates. In addition, the continuous metal network is expected to act as a conductive matrix, providing a rapid supply of electrons to the catalytic sites, which, in turn, increases the efficiency of the electrochemical reaction.

The crystal phase structure of the samples was examined by XRD (Figure 2C). The XRD patterns and EDX spectra of the studied catalytic layers are almost identical, since polycrystalline nickel predominates in their composition (Figure 2B,C). The EDX spectrum shows the lines associated with nickel: L_α1_ = 0.85 keV, K_α1_ = 7.48 keV, and K_β1_ = 8.27 keV (Figure 2B).

### 2.2. Linear Sweep Voltammetry

**Figure 3:**
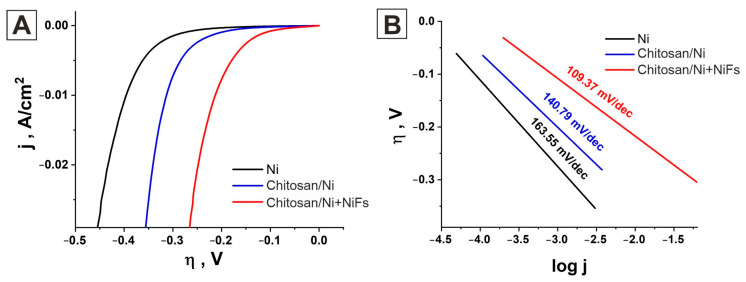
Cathodic polarization curves for the ***Chitosan/Ni*** and ***Chitosan/Ni + NiFs*** electrocatalysts, obtained in an aqueous 0.5 M H_2_SO_4_ solution at a scan rate of 2 mV/s. The black line represents the response of the bare ***Ni*** catalyst (**A**). Tafel curves for the ***Chitosan/Ni*** and ***Chitosan/Ni + NiFs*** electrocatalysts (**B**). The black curve corresponds to the bare ***Ni*** catalyst. The curves are given with iR compensation; scan rate = 2 mV/s.

To study the electrocatalytic activity of the obtained layers in the hydrogen evolution reaction, linear sweep voltammetry (LSV) studies were conducted. Figure 3A shows the polarization curves for all studied electrocatalysts, recorded in an aqueous 0.5 M H_2_SO_4_ solution at a scan rate of 2 mV/s.

As shown in Figure 3, the electrocatalytic activity of the studied coatings differs significantly. Among the presented electrocatalysts, ***Chitosan/Ni + NiFs*** exhibits the highest efficiency in the hydrogen evolution reaction. The HER overpotential at 10 mA/cm^2^ for this system is 0.213 V. The efficiency of the ***Chitosan/Ni*** coating is lower than that of ***Chitosan/Ni + NiFs***. The HER overpotential for this electrode is 104 mV higher, amounting to 0.317 V. For comparison, the figure also shows the polarization curve of the ***Ni*** layer. The overpotential on the Ni layer is 0.396 V, which is 183 mV higher than that for ***Chitosan/Ni + NiFs***. The kinetics of the hydrogen evolution reaction for all electrocatalysts were studied using Tafel curve analysis. For this purpose, the polarization curves shown in Figure 3 are presented in semilogarithmic coordinates (Figure 3B). Key parameters such as the Tafel slope (*b*), charge transfer coefficient (α), and exchange current density (*j_o_*) were obtained by analyzing the Tafel curves. The Tafel slope for the ***Ni*** layer is 163.55 mV/dec. According to the mechanism of the hydrogen evolution reaction in acidic media [56], this Tafel slope value indicates that the rate-limiting step in the ***Ni*** layer is the ion discharge step with the formation of adsorbed hydrogen atoms (the Volmer step). The charge transfer coefficient and exchange current density values for this electrocatalyst are 0.361 and 2.09 × 10^−5^ A/cm^2^, respectively (Table 1). For the ***Chitosan/Ni*** electrocatalyst, similar values of the Tafel slope (140.79 mV/dec) and charge transfer coefficient (0.419) were obtained. However, according to Figure 3A, the overpotential of the hydrogen evolution reaction at 10 mA/cm^2^ on this electrode is 20% lower than that on a nickel layer electrodeposited on the surface of a glassy carbon electrode in the absence of a chitosan layer. In addition, the current density at zero overpotential obtained on ***Chitosan/Ni*** is 1.8 times higher than that on ***Ni***. Although the same material, nickel, was used as the catalytic layer in both cases, according to the above data, their electrocatalytic activities differ significantly. We attribute the increased activity of the ***Chitosan/Ni*** electrocatalyst to its increased specific surface area. As shown via AFM, ***Chitosan/Ni*** has a higher roughness than ***Ni***. Analysis of the polarization curves revealed significant differences in the kinetic parameters of the ***Chitosan/Ni + NiFs*** catalyst. For this system, the Tafel slope is 109.37 mV/dec, confirming that the hydrogen evolution reaction occurs via the Volmer-Heyrovsky mechanism with ion discharge as the rate-limiting stage. The key difference for this catalyst is the increase in the charge transfer coefficient to 0.539 versus 0.361 for pure ***Ni***, indicating acceleration of the initial stage of the reaction (Volmer stage). An investigation of the HER kinetics for all electrocatalysts revealed that the charge transfer coefficient increases in the series ***Ni***, ***Chitosan/Ni***, and ***Chitosan/Ni + NiFs***, with values of 0.361, 0.419, and 0.539, respectively (Table 1). The increase in the charge transfer coefficient to 0.539 for the nickel fibers indicates a reduction in the energy barrier of the electrochemical reaction and an enhancement in the electron transfer rate. This is a key factor for improving the functional characteristics of catalysts in electrochemical processes. The improved charge transfer in the anisotropic network of oriented nickel fibers is primarily due to its ordered morphology. Due to their structure, the oriented fibers create continuous pathways, ensuring directed charge transport. This approach minimizes the resistance at grain boundaries, in contrast to isotropic nanoparticles or films. Furthermore, compared to a pure nickel film, the network of oriented nickel fibers is an anisotropic structure with a high specific surface area. Such a surface provides a larger contact area with the electrolyte, reducing the local charge density. In addition, the exchange current density j_0_ (current at zero overpotential) for ***Chitosan/Ni + NiFs*** catalyst is 5 times higher than for pure ***Ni*** (Table 1). This means that the ***Chitosan/Ni + NiFs*** catalytic system provides a fivefold increase in the hydrogen evolution reaction rate.

We carried out studies on ***Chitosan/NiFs*** (without a ***Ni*** sublayer), but the results were completely unsatisfactory. Unfortunately, it is impossible to achieve good adhesion of ***NiFs*** on a clean chitosan surface, leading to the destruction of the upper layer (nickel fiber network) immediately after placement in the electrolyte. Moreover, deposition of the ***NiFs*** network on the surface of pure chitosan causes a change in the morphology of the polymer film (film swelling and the formation of wrinkled areas). These outcomes forced us to exclude the results from this work, as they were incorrect. A good alternative is the creation of a nickel sublayer, on which the ***NiFs*** network is well deposited.

Another way to compare the efficiency of catalysts in the hydrogen evolution reaction is to compare the resulting current density at a fixed overpotential. Table 1 presents the current densities j for all electrocatalytic layers at an overpotential of −150 mV. According to the data, ***Chitosan/Ni + NiFs*** provides the best electrocatalytic activity. The current density at a fixed overpotential for this system is 16 times higher than that of pure Ni. Based on the data obtained from the polarization curves and Tafel curves, it can be concluded that the use of chitosan as a matrix for the deposition of nickel and nickel fibers leads to improved electrocatalytic activity and an increase in the hydrogen evolution reaction rate, as compared to the bare ***Ni*** catalyst. However, it is worth noting that the Tafel curves presented in Figure 3B are normalized to the geometric area. Therefore, the above results do not allow us to draw a definitive conclusion as to whether the observed improvement in the catalytic activity of the ***Chitosan/Ni*** and ***Chitosan/Ni + NiFs*** systems is due to an increase in their specific surface area or a change in their intrinsic catalytic activity. To determine this, it is necessary to estimate the actual surface area of the electrocatalysts. For this purpose, we chose electrochemical impedance spectroscopy (EIS).

### 2.3. Electrochemical Impedance Spectroscopy

**Figure 4:**
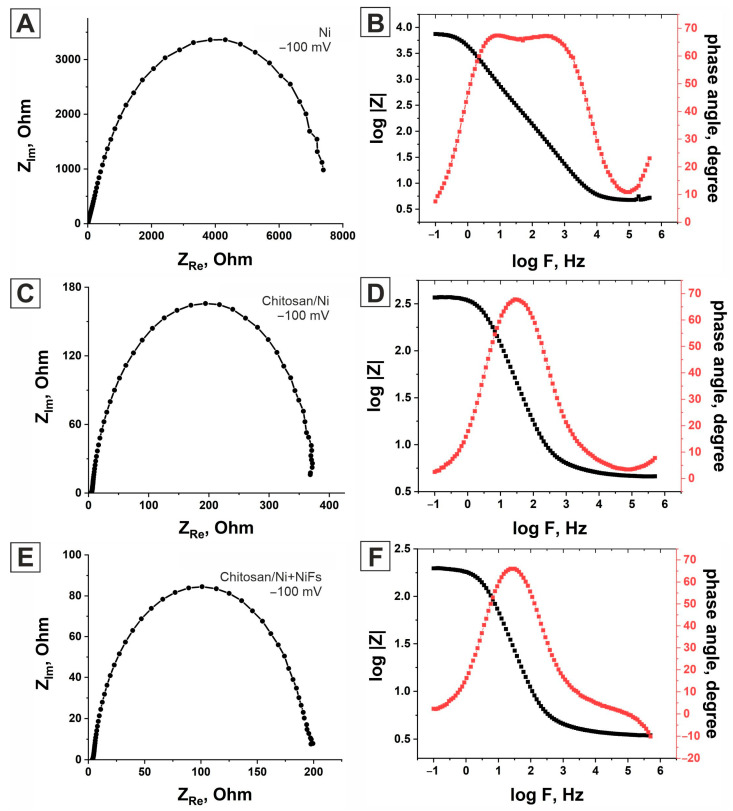
Nyquist and Bode plots for electrocatalysts based on ***Ni*** (**A**,**B**), ***Chitosan/Ni*** (**C**,**D**), and ***Chitosan/Ni + NiFs*** (**E**,**F**) at an overpotential of −100 mV. The dots correspond to experimental data, and the solid lines correspond to simulated data.

To obtain complete information about the electrode/electrolyte interface and the processes occurring on the surface of the studied catalysts, we recorded the electrochemical impedance spectroscopy (EIS) curves in the frequency range from 0.1 Hz to 500 kHz at various overpotentials. Figure 4 shows the EIS spectra for ***Ni*** (Figure 4A), ***Chitosan/Ni*** (Figure 4C), and ***Chitosan/Ni + NiFs*** (Figure 4E) at an overpotential of −100 mV relative to the equilibrium potential. The spectra are presented as Nyquist and Bode curves (Figure 4B,D,F). According to the research results, only one time constant is characteristic of all catalysts. The electrochemical impedance spectra were then simulated using a specialized equivalent electrical circuit. In Figure 4, the symbols correspond to the experimental data, and the solid curves correspond to the simulated ones.

**Figure 5:**
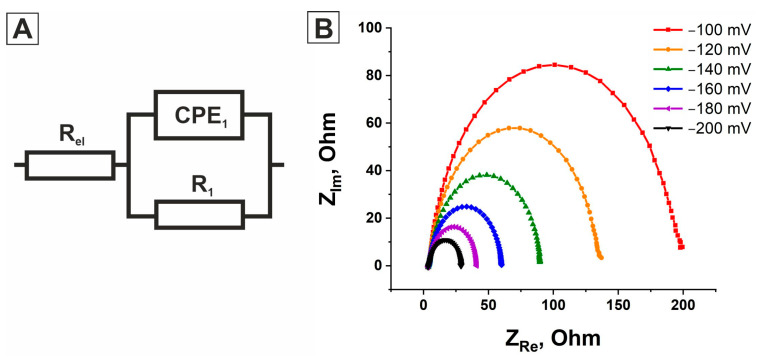
The electrical equivalent circuit (EEC) model used to describe the hydrogen evolution reaction on the studied electrocatalysts (**A**), and a Nyquist plot showing the electrochemical response of the ***Chitosan/Ni + NiFs*** system at different overpotentials. The dots on the curves correspond to experimental data, and the solid lines correspond to simulated data (**B**).

To simulate the EIS spectra of all electrocatalysts, we used the electrical equivalent circuit (EEC) (a single-time constant model) shown in Figure 5A. Figure 4 shows that using this model to describe the electrochemical impedance response of the ***Ni***, ***Chitosan/Ni***, and ***Chitosan/Ni + NiFs*** electrocatalysts yields the best fit between the simulated and experimental data. Furthermore, it is worth noting that the best fit between the simulated and experimental data is achieved when the capacitance in the EEC is replaced by a constant-phase element (CPE). The constant-phase element is known to model the behavior of a non-ideal capacitor.

To determine whether the time constant in the EEC is related to HER kinetics or to the nature of the catalyst itself, the EIS spectra for all electrocatalytic layers were recorded at different overpotentials in the potential range from equilibrium potential (EP) to (EP + 200 mV). The recorded EIS spectra for the ***Chitosan/Ni + NiFs*** system are shown in Figure 5B. Analysis of the electrochemical impedance spectra for this system, obtained at different overpotentials, revealed a consistent decrease in the diameter of the semicircles in the Nyquist diagrams with increasing overpotential. Modeling of the spectra using an equivalent electrical circuit demonstrated a decrease in both the process time constant and the charge transfer resistance (R_1_), while the CPE value remained stable within 308 ± 14 μF∙S^n−1^∙cm^−2^ (Table 2). This dependence of the kinetic parameters on the potential indicates that the time constant is related to the electrochemical hydrogen evolution stage, rather than to the material properties. The obtained results are consistent with those from the Tafel analysis, which confirmed that the rate of hydrogen evolution on the ***Chitosan/Ni + NiFs*** catalyst surface is limited by the ion discharge stage (electron transfer). A similar pattern was observed for the ***Ni*** and ***Chitosan/Ni*** electrocatalysts. The time constant also decreased with increasing overpotential (Figures S1 and S2). The electrical equivalent circuit parameters for the ***Ni*** and ***Chitosan/Ni*** electrocatalysts as a function of overpotential are presented in Table 2. As can be seen from the table, with increasing overpotential, the value of R_1_ decreases, while the value of the constant-phase element remains virtually unchanged (for ***Chitosan/Ni***, 204.68 ± 11 μF∙S^n−1^∙cm^−2^; for ***Ni***, 28.15 ± 1 μF∙S^n−1^∙cm^−2^). Accordingly, as in the case of ***Chitosan/Ni + NiFs***, the time constant in the EEC is related to the kinetics of the HER.

In addition to providing information on electrochemical activity, EIS allows the real electrochemically active area S_real_ of the catalysts to be determined. We calculated the real electrochemically active area of the studied systems using the capacitance of the electrical double layer (C_DL_) and the formula S_real_ = C_DL_/20 F∙cm^−2^. C_DL_ can be calculated using the constant-phase element CPE, the value of which is determined during the selection of the EEC parameters. Knowing S_real_, one can calculate the catalyst surface roughness coefficient σ = S_real_/S_geometric_. The calculated values of the double-layer capacitance (C_DL_) over the entire studied overpotential range are presented in Table 3. According to the presented data, the capacity of the studied catalysts remains almost unchanged over the studied overpotential range. ***Chitosan/Ni + NiFs*** has the highest capacity. This means that the hydrogen evolution reaction on the surface of this catalyst proceeds faster and more efficiently. Table 3 also presents the surface roughness values σ for the studied catalysts. In order to determine whether the increased electrocatalytic activity of the ***Chitosan/Ni*** and ***Chitosan/Ni + NiFs*** layers is due to an increase in their specific surface area or a change in their intrinsic catalytic activity, we normalized the polarization curves by the surface roughness coefficient. Table 3 presents the current densities (j/σ) normalized by the roughness coefficient for all electrocatalysts at an overpotential of −150 mV. As can be seen from the table, the control nickel electrode has the highest normalized j/σ value. However, data based on the geometric area (Table 1) show that ***Ni*** exhibits the lowest efficiency in the hydrogen evolution reaction in the series under consideration. Thus, the data in Table 3 allow us to conclude that the increased electrocatalytic activity of ***Chitosan/Ni*** and ***Chitosan/Ni + NiFs***, as expected, is associated with the developed morphology of their surface and, as a result, their larger specific surface area. The developed morphology is achieved through the use of a complex structure of the catalytic layer: a biopolymer matrix of chitosan, a layer of nickel nanoparticles, and a layer comprising an anisotropic network of nickel fibers.

### 2.4. Activation Energy

**Figure 6:**
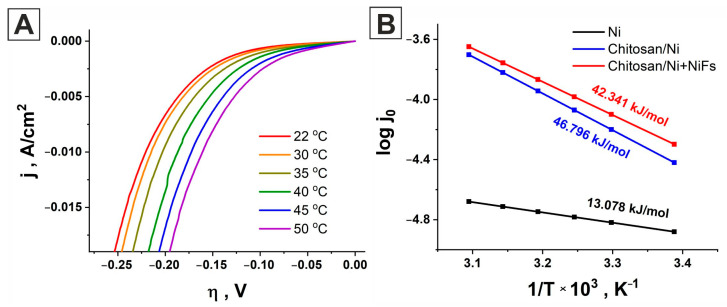
Cathodic polarization curves for the ***Chitosan/Ni + NiFs*** electrocatalyst obtained at different temperatures in an aqueous 0.5 M H_2_SO_4_ solution at a scan rate of 2 mV/s (**A**); the dependence of the logarithm of the exchange current density on the inverse temperature for ***Ni*** (black line), ***Chitosan/Ni*** (blue line), and ***Chitosan/Ni + NiFs*** (red curve) (**B**).

To quantitatively assess the effect of temperature on the rate of hydrogen evolution on the studied electrocatalysts, polarization curves were recorded in the temperature range of 303–323 K. Figure 6A shows the measurement results for the ***Chitosan/Ni + NiFs*** catalytic system, obtained at various temperatures. As expected, the current density also increases with increasing temperature. Similar behavior was observed for the ***Ni*** and ***Chitosan/Ni*** systems (Figures S3 and S4).

Figure 6B shows the dependence of the logarithm of the exchange current density, log j_0_, on the reciprocal temperature, 1/T⋅10^3^, for all studied systems. As can be seen from the figure, the dependences are linear in a semi-logarithmic plot, which is in accordance with the Arrhenius law:

log j_0_ = −E_act_/2.3RT + logA_0_, (1)

where T is the temperature, R is the universal gas constant, and A_0_ is the pre-exponential factor. According to this equation, the activation energy of the systems at zero overpotential can be calculated as the slope of the line. The calculated activation energy values, E_act_, for each system are presented in Figure 6B. The obtained data are in good agreement with the EIS data (Table 3). The pure nickel electrocatalyst (bare ***Ni***) has a lower activation energy (13.078 kJ/mol). The calculated E_act_ values show that the use of chitosan and a network of oriented nickel fibers significantly alters the process kinetics, leading to an increase in the activation energy. The activation energy for ***Chitosan/Ni*** is 46.796 kJ/mol, while for ***Chitosan/Ni + NiFs***, it is 42.341 kJ/mol. This demonstrates the significant influence of the modified coatings on charge transfer processes and electrode reactions. It is clear that using chitosan as a matrix and nickel fibers as an additional layer increases the reaction energy barrier, although the exchange current increases. The obtained data once again demonstrate the possibility of controlling the electrochemical activity of catalysts through surface modification. In this work, the influence of both the activation energy value and surface morphology on performance was investigated. Both of these factors have a significant impact on the catalyst’s ultimate efficiency. This is especially noticeable for the ***Chitosan/Ni + NiFs*** system: the activation energy is slightly higher than that for ***Chitosan/Ni***, but due to the high electrochemically active surface area (ECSA), the overall performance of the ***Chitosan/Ni + NiFs*** system is better.

### 2.5. Gas Chromatography

**Figure 7:**
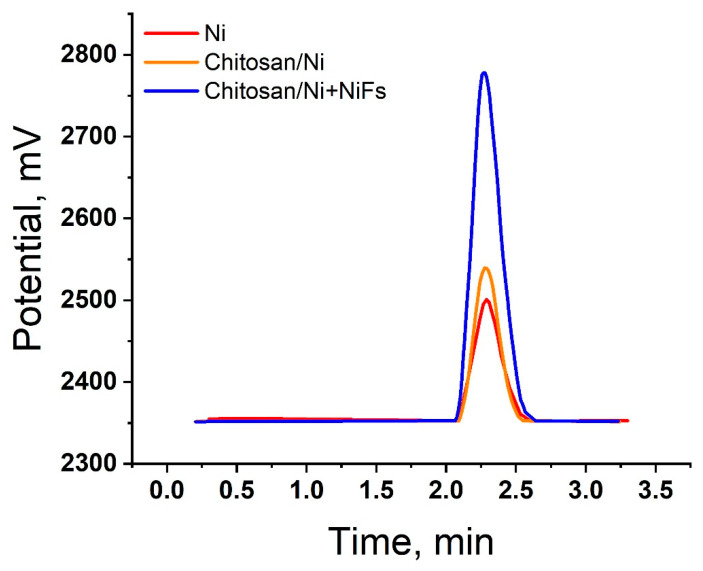
Chromatograms characterizing the evolution of hydrogen on ***Ni***, ***Chitosan/Ni***, and ***Chitosan/Ni + NiFs***.

To confirm the electrocatalytic release of molecular hydrogen on the studied electrocatalysts, a chromatographic analysis was performed. Figure 7 shows chromatograms recorded 1800 s after the start of electrolysis. A characteristic peak corresponding to gaseous H_2_ was observed for all studied systems. The highest signal intensity, reflecting the maximum amount of hydrogen released, was recorded for the ***Chitosan/Ni + NiFs*** composite material, which is consistent with the electrochemical measurement data.

The lowest amount of hydrogen was recorded for the ***Ni*** system. The introduction of a chitosan matrix (***Chitosan/Ni***) increased the hydrogen yield, as compared to pure ***Ni***. The highest signal intensity, reflecting the maximum amount of hydrogen released, was recorded for the ***Chitosan/Ni + NiFs*** composite material. Thus, the chromatographic analysis results correlate with the electrochemical measurements, where the ***Chitosan/Ni + NiFs*** system demonstrates the best kinetic parameters among those in the considered set.

### 2.6. Stability Test

The hierarchical structure of ***Chitosan/Ni + NiFs***, consisting of a biopolymer (chitosan) with a layer of nickel nanoparticles and an oriented network of ***Ni*** fibers, improves catalytic efficiency through the following factors: a significant increase in the specific surface area and accessibility of active sites due to the spiked structure, and improved charge transfer due to the high specific electrical conductivity of the network. Good adhesion of the polymer film to the glassy carbon and the network structure of the top layer ensure mechanical stability and prevent the aggregation of active sites. This ensures that the material maintains high catalytic activity and stable performance over a long period of time, as confirmed through stability tests.

**Figure 8:**
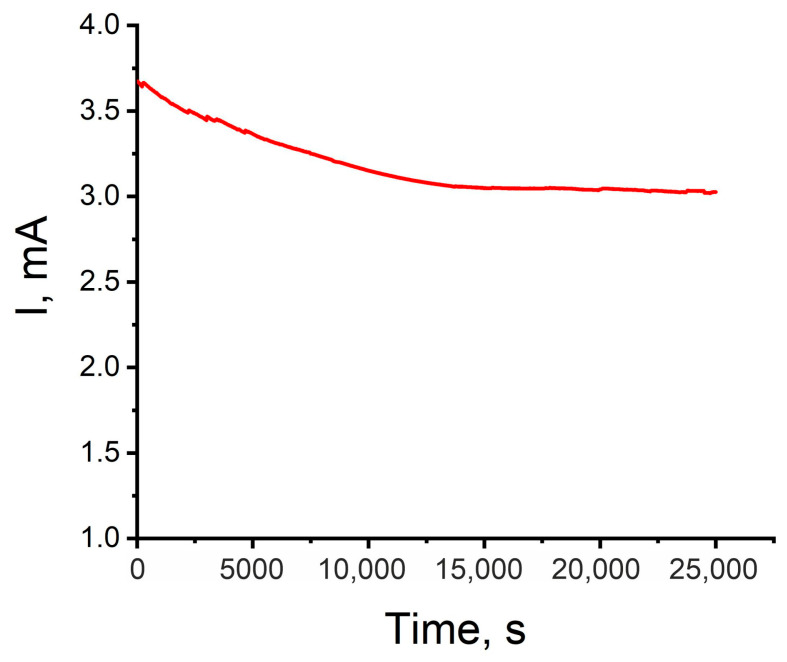
Long-term stability of ***Chitosan/Ni + NiFs*** at a fixed potential of −0.558 V (vs. Ag/AgCl).

Figure 8 shows a chronoamperometric curve characterizing the stability of the ***Chitosan/Ni + NiFs*** electrocatalyst in the hydrogen evolution reaction. The measurements were performed at a constant potential of −0.558 V (vs. Ag/AgCl) in 0.5 M H_2_SO_4_ for 25,000 s. As can be seen from the plot, after the establishment of a steady-state regime, the current exhibited stable behavior, remaining practically unchanged throughout the entire experiment. The absence of pronounced current decay indicates the high stability of ***Chitosan/Ni + NiFs*** under conditions of long-term electrocatalytic hydrogen evolution.

## 3. Experimental Section

The electrocatalytic activity of the layers in the hydrogen evolution reaction was studied in a 0.5 M H_2_SO_4_ solution (pH = 0) within the temperature range of 298–303 K. All electrochemical studies were carried out in a standard three-electrode cell in an oxygen-free environment, which was achieved by continuously purging the electrolyte with argon. A graphite electrode was used as an auxiliary electrode, and a saturated silver chloride (Ag/AgCl) electrode served as a reference electrode. Catalytic layers based on chitosan and nickel were formed on a glassy carbon electrode with an area of 0.25 cm^2^. The technique included the deposition of a chitosan film on the glassy carbon (GC) surface from a 0.1% solution in acetic acid, followed by the electrodeposition of nickel from a 0.7 M NiCl_2_∙6H_2_O solution at 24 mA/cm^2^. Electrodeposition was carried out in an argon atmosphere. The electrocatalytic layer obtained in this way is referred to herein as ***Chitosan/Ni***. To obtain the ***Chitosan/Ni + NiFs*** system, nickel fibers [61,62] were deposited onto a pre-existing ***Chitosan/Ni*** layer. The fibers were deposited in an oriented network in the presence of a magnetic field. A bare ***Ni*** sample was deposited directly onto glassy carbon from a 0.7 M NiCl_2_∙6H_2_O solution at 24 mA/cm^2^. All reagents were purchased from Sigma-Aldrich (St. Louis, MO, USA).

Polarization curves were recorded on a P-45X potentiostat at 2 mV/s, taking into account iR compensation. Electrochemical impedance measurements were performed using a P-45X potentiostat–galvanostat (Electrochemical instruments, Chernogolovka, Russia) equipped with a frequency response analysis (FRA) module. The studies were conducted in a three-electrode electrochemical cell at room temperature (25 ± 3 °C) in the frequency range from 500 kHz to 0.1 Hz. Impedance curves for the studied systems were recorded at fixed overpotentials in the potential range from equilibrium potential (EP) to (EP + 200 mV). The surface topography of the electrocatalysts was examined using atomic force microscopy and optical confocal laser scanning microscopy. AFM images were obtained using a MultiMode V atomic force microscope (Veeco, Plainview, New York, NY, USA) in tapping mode. To obtain reliable information, surface scanning was performed over various areas. The measurement parameters included scanned area sizes from 1 × 1 μm^2^ to 10 × 10 μm^2^, a resolution of 512 × 512 pixels, and a scanning speed of 0.5–1.5 Hz. Optical images were obtained using a Leica DCM3D confocal microscope (Leica, Wetzlar, Germany). To confirm the evolution of hydrogen gas at the developed electrodes, gas chromatography investigations were carried out in a sealed three-electrode electrochemical cell. The electrolyte was a 0.5 M H_2_SO_4_ solution, pre-saturated with argon to remove dissolved oxygen. Before measurements, the chromatographic system was calibrated using standard gas mixtures of hydrogen in nitrogen. Analysis was performed on a Crystallux-4000M chromatograph (Meta-chrome, Yoshkar-Ola, Russia), using high-purity argon (99.999%) as the carrier gas. Gas-tight 1 mL syringes (Hamilton, Reno, NV, USA) were used for sampling. During the experiment, a constant potential of −0.6 V relative to the silver chloride electrode was applied to the working electrode, corresponding to the hydrogen evolution reaction region for all studied electrocatalysts. Gas samples were collected from the cell’s gas space 1800 s after the start of electrolysis. Transmission electron microscopy (TEM) and TEM-EDX experiments were performed on a Hitachi HT7700 (Hitachi, Tokyo, Japan) (wolfram filament, accelerating voltage = 100 kV, Thermo Scientific energy-dispersive X-ray detector (Waltham, MA, USA)). Powder X-ray diffraction patterns were obtained on a Shimadzu XRD-7000 automatic X-ray diffractometer (Shimadzu, Kyoto, Japan) in X-ray tube operation mode, with an accelerating voltage of 40 kV and a tube current of 30 mA.

## 4. Conclusions

This work involved the development and comprehensive study of electrocatalysts based on a chitosan biopolymer matrix and an anisotropic network of oriented nickel fibers for the hydrogen evolution reaction. It was found that the combined use of the chitosan framework and the anisotropic nickel fiber network significantly increased the specific surface area of the catalyst. This occurred due to the formation of a hierarchical structure with developed surface roughness, as confirmed via atomic force microscopy and optical microscopy. A direct relationship between the surface morphology and catalytic activity was revealed. The ***Chitosan/Ni + NiFs*** electrocatalyst was shown to reduce the hydrogen evolution overpotential to 213 mV at a current density of 10 mA/cm^2^. Furthermore, it significantly lowered the charge transfer resistance, which overall ensured higher efficiency compared to ***Ni*** and ***Chitosan/Ni***. The effectiveness of the ***Chitosan/Ni + NiFs*** system was also confirmed by chromatographic measurements, in which this electrocatalyst demonstrated the best hydrogen production performance. The results confirm the promise of combining a biopolymer matrix with an anisotropic network of oriented nickel fibers for the creation of efficient electrocatalysts, which opens up new opportunities for the development of environmentally safe hydrogen production systems involving electrolysis.

## Figures and Tables

**Table 1:** HER kinetic parameters (*b*, *j_o_*, *α*) obtained by analyzing the Tafel curves shown in Figure 3B. To enable comparisons of the electrocatalytic activity of the studied catalysts, the table also presents the overpotential η required to achieve a current density of 10 mA/cm^2^ and the current density at a fixed overpotential value (−150 mV).

Catalyst	b, mV/dec	j_o_, A/cm^2^	α	η, mV at 10 mA/cm^2^	j, μA/cm^2^ at −150 mV
Ni	163.55	2.09 × 10^−5^	0.361	−396	163.5
Chitosan/Ni	140.78	3.76 × 10^−5^	0.419	−317	405.56
Chitosan/Ni + NiFs	109.37	10.308 × 10^−5^	0.539	−213	2608
Pt-Tb/C [57]	23.3	-	-	−24	-
Ni-PGE [58]	208	-	-	−210	-
Se12%-NiTe_2_ [59]	38	-	-	375	-
Ni-W Films [60]	186	-	-	363	-

**Table 2:** Parameters of the electrical equivalent circuit for the HER with the studied electrocatalysts.

	Ni		Chitosan/Ni		Chitosan/Ni + NiFs	
Overpotential, mV	R_1_, Ohm	CPE, μF∙S^n−1^∙cm^−2^	R_1_, Ohm	CPE, μF∙S^n−1^∙cm^−2^	R_1_, Ohm	CPE, μF∙S^n−1^∙cm^−2^
100	8691.4	27.76	373	174	193.56	294.02
120	4473.4	27.96	203.13	191.29	131.45	299.11
140	2913	27.023	124.19	193.28	86.044	305.75
160	1878	27.385	76.076	204.85	56.191	304.42
180	1270	29.369	47.035	219.95	37.23	317.18
200	766	29.43	30.406	244.75	25.121	332.32

**Table 3:** Double-layer capacitance C_DL_ (normalized by the geometric electrode area) and surface roughness σ of the studied electrocatalysts, calculated based on EIS data over the studied voltage range.

Catalyst	C_DL_, F/cm^2^	σ	j/σ, μA/cm^2^ (at −150 mV)
Ni	3.5 × 10^−5^	1.6	102
Chitosan/Ni	42.4 × 10^−5^	21.2	19
Chitosan/Ni + NiFs	66.8 × 10^−5^	33.4	78

## Data Availability

The original contributions presented in this study are included in the article/Supplementary Materials. Further inquiries can be directed to the corresponding author.

## Supplementary Materials

The following supporting information can be downloaded at: https://www.mdpi.com/article/10.3390/molecules31010150/s1, Figure S1. Nyquist plot showing the electrochemical response of a Ni-based catalyst at different overpotentials. Symbols on the curves represent experimental data, and solid lines represent simulated data; Figure S2. Nyquist plot showing the electrochemical response of the ***Chitosan/Ni*** system at different overpotentials. Symbols on the curves represent experimental data, and solid lines represent simulated data; Figure S3. Cathodic polarization curves of the Ni-based electrocatalyst, obtained at different temperatures in an aqueous solution of 0.5 M H_2_SO_4_ at a scan rate of 2 mV/s. The curves are given taking into account iR compensation; Figure S4. Cathodic polarization curves of the ***Chitosan/Ni*** electrocatalyst, obtained at different temperatures in an aqueous solution of 0.5 M H_2_SO_4_ at a scan rate of 2 mV/s. The curves are given taking into account iR compensation; Figure S5. Optical images of the oriented network of nickel fibers deposited on ***Chitosan/Ni*** (***Chitosan/Ni + NiFs***); Figure S6. The surface images of ***Chitosan/Ni + NiFs*** obtained by 3D confocal microscopy; Figures S7–S10. TEM image of ***NiFs***.

## Abbreviations

The following abbreviations are used in this manuscript:

HER	Hydrogen evolution reaction
AFM	Atomic force microscopy
LSV	Linear sweep voltammetry
EIS	Electrochemical impedance spectroscopy
EEC	Electrical equivalent circuit
CPE	Constant-phase element
EP	Equilibrium potential
FRA	Frequency response analysis
TEM	Transmission electron microscopy
EDX	Energy-Dispersive X-ray spectroscopy
XRD	X-Ray Diffraction
GC	Glassy carbon
