# Supplementary material for: A Biopolymer System Based on Chitosan and an Anisotropic Network of Nickel Fibers in the Hydrogen Evolution Reaction
Source: Molecules. 2026 Jan 1;31(1):150. doi: 10.3390/molecules31010150 (PMC12787352; doi:10.3390/molecules31010150)
Supplement: Supplementary file 1 [file molecules-31-00150-s001.zip › molecules-4010402-supplementary.pdf]

Supplementary Materials

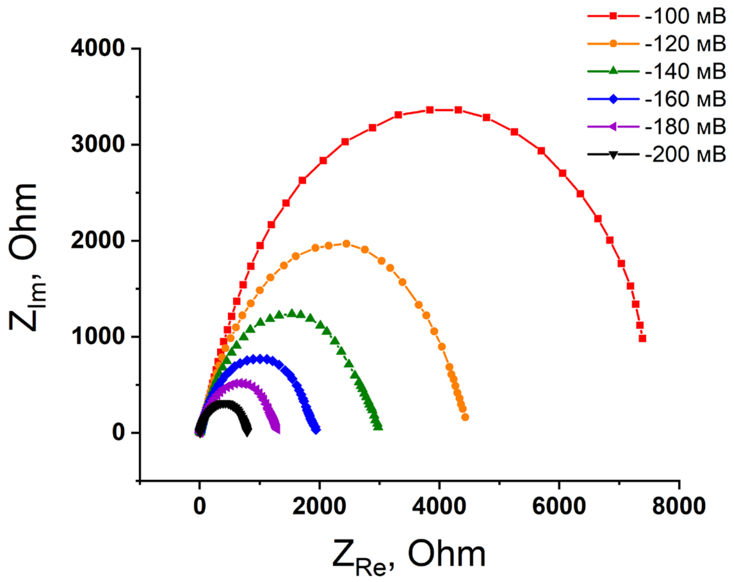

Figure S1. Nyquist plot showing the electrochemical response of a ***Ni***-based catalyst at different overpotentials. Symbols on the curves represent experimental data, and solid lines represent simulated data

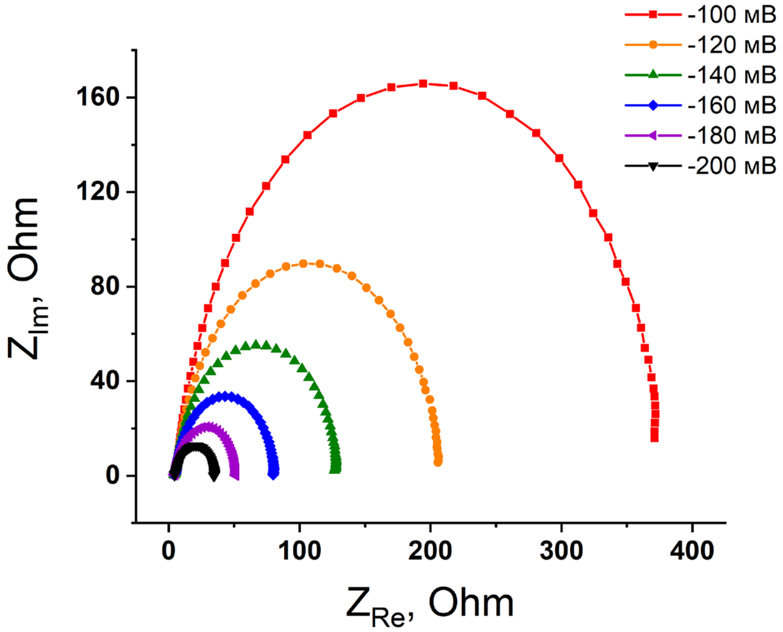

Figure S2. Nyquist plot showing the electrochemical response of the ***Chitosan/Ni*** system at different overpotentials. Symbols on the curves represent experimental data, and solid lines represent simulated data

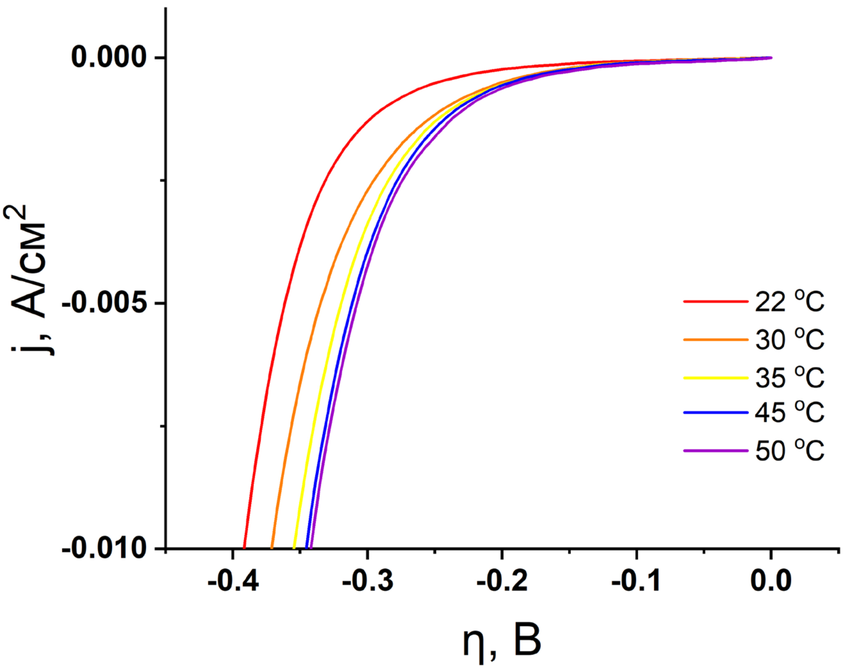

Figure S3. Cathodic polarization curves of the ***Ni***-based electrocatalyst, obtained at different temperatures in an aqueous solution of 0.5 M H_2SO_4 at a scan rate of 2 mV/s. The curves are given taking into account iR compensation

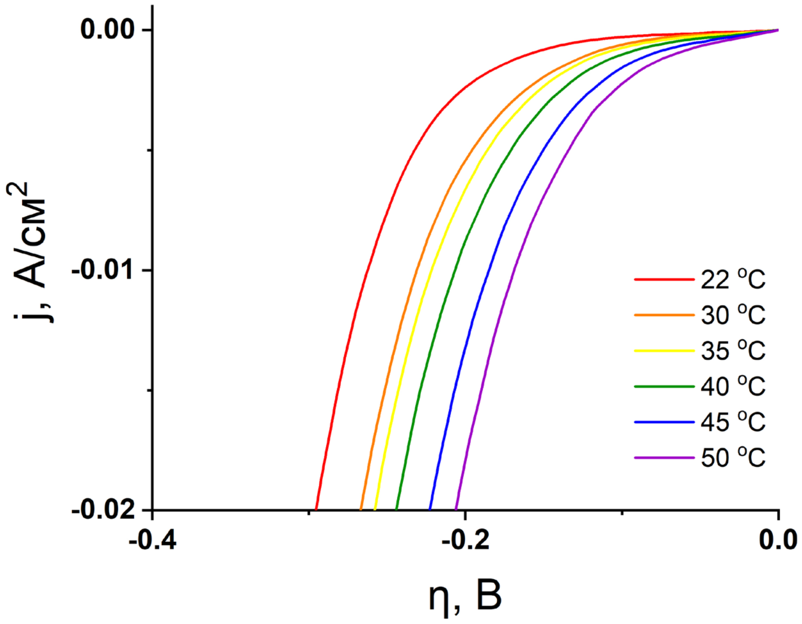

Figure S4. Cathodic polarization curves of the ***Chitosan/Ni*** electrocatalyst, obtained at different temperatures in an aqueous solution of 0.5 M H_2SO_4 at a scan rate of 2 mV/s. The curves are given taking into account iR compensation

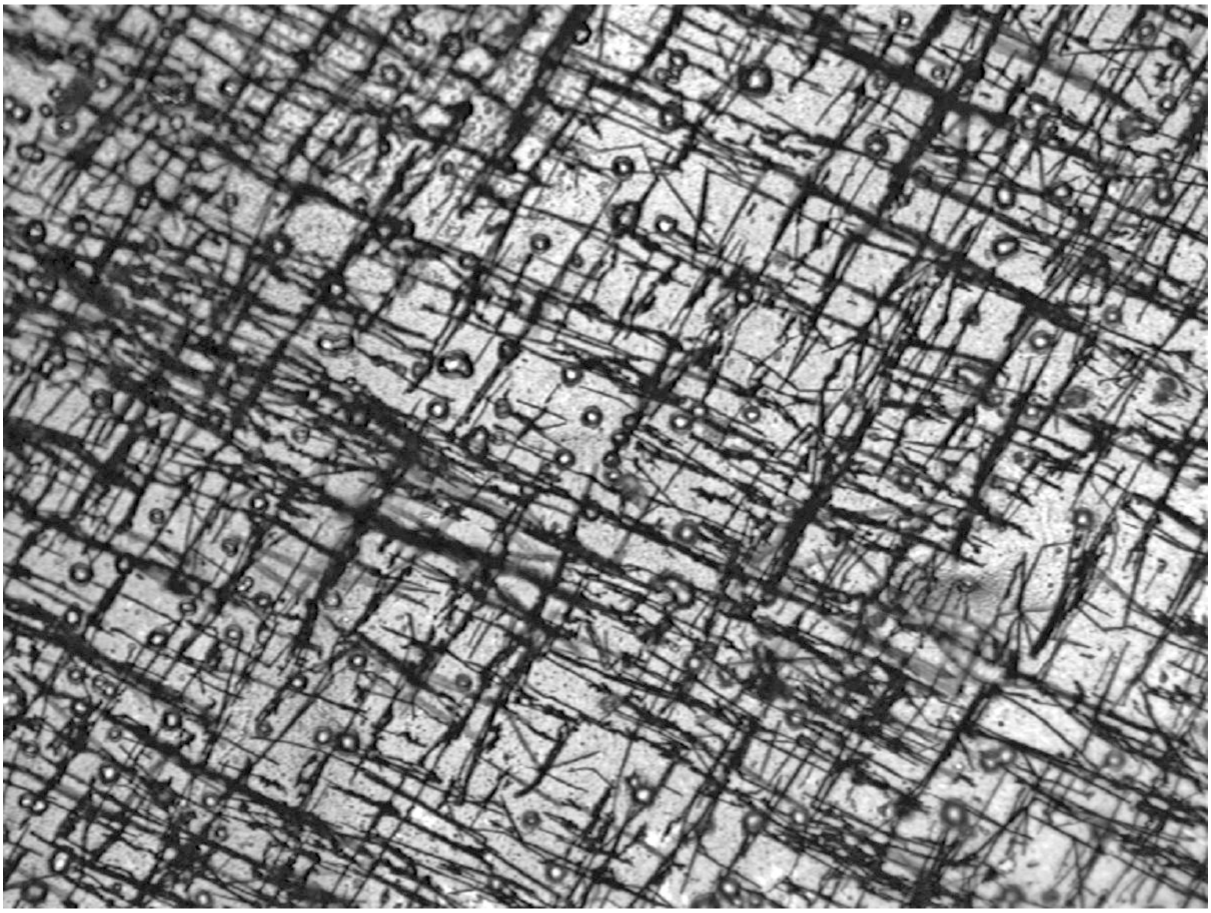

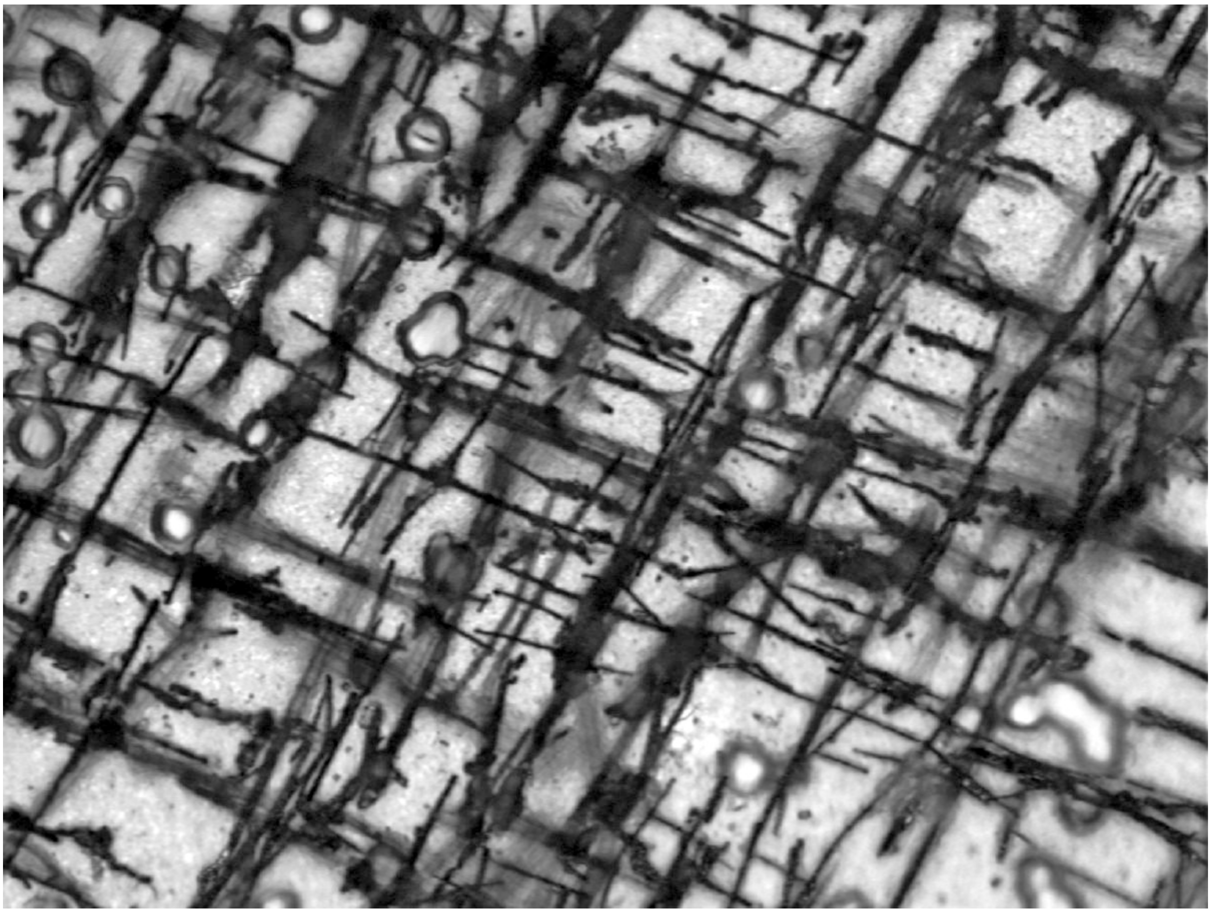

Figure S5. Optical images of the oriented network of nickel fibers deposited on ***Chitosan/Ni*** (***Chitosan/Ni+NiFs***)

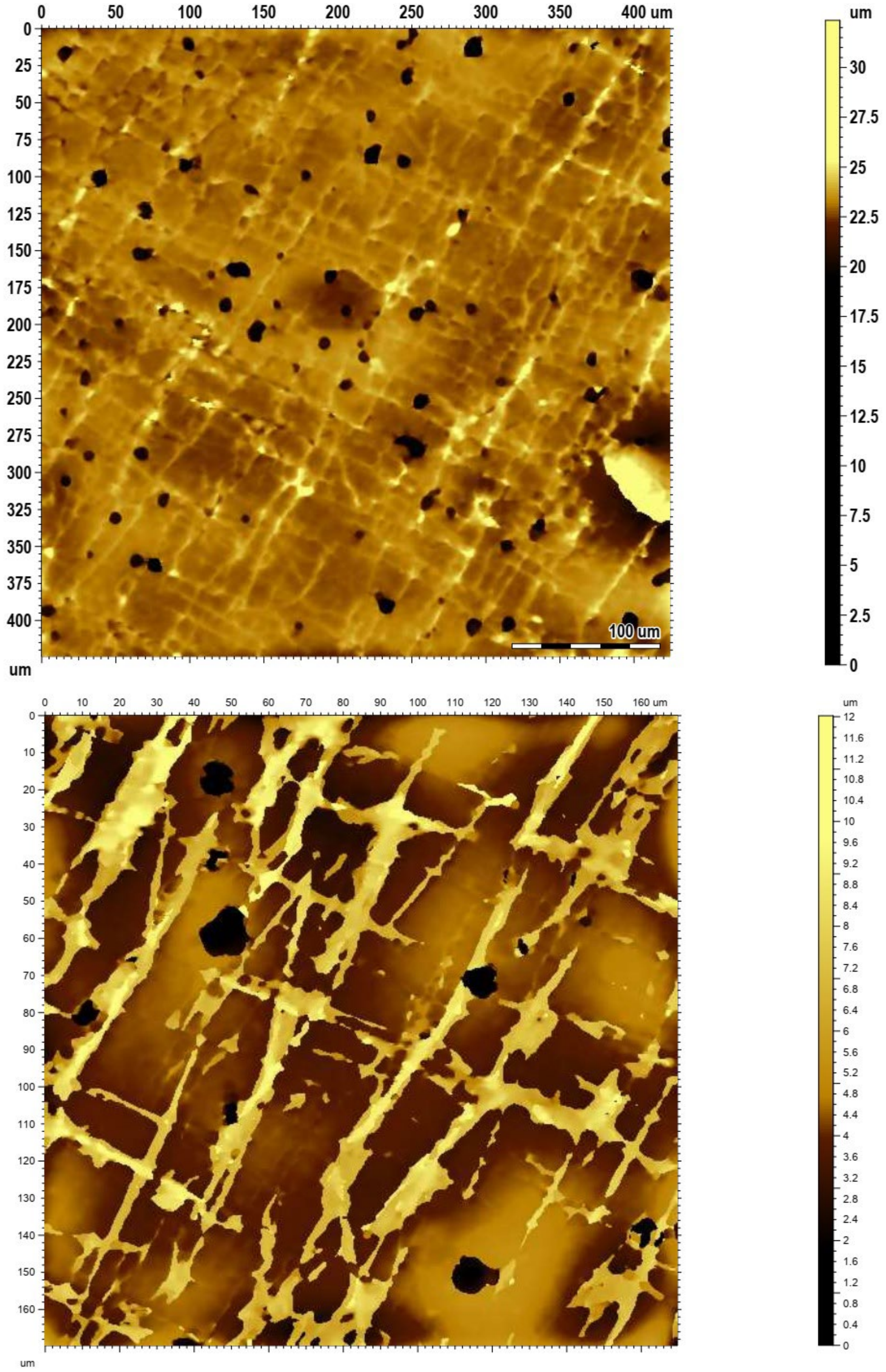

Figure S6. The surface images of ***Chitosan/Ni+NiFs*** obtained by 3D confocal microscopy

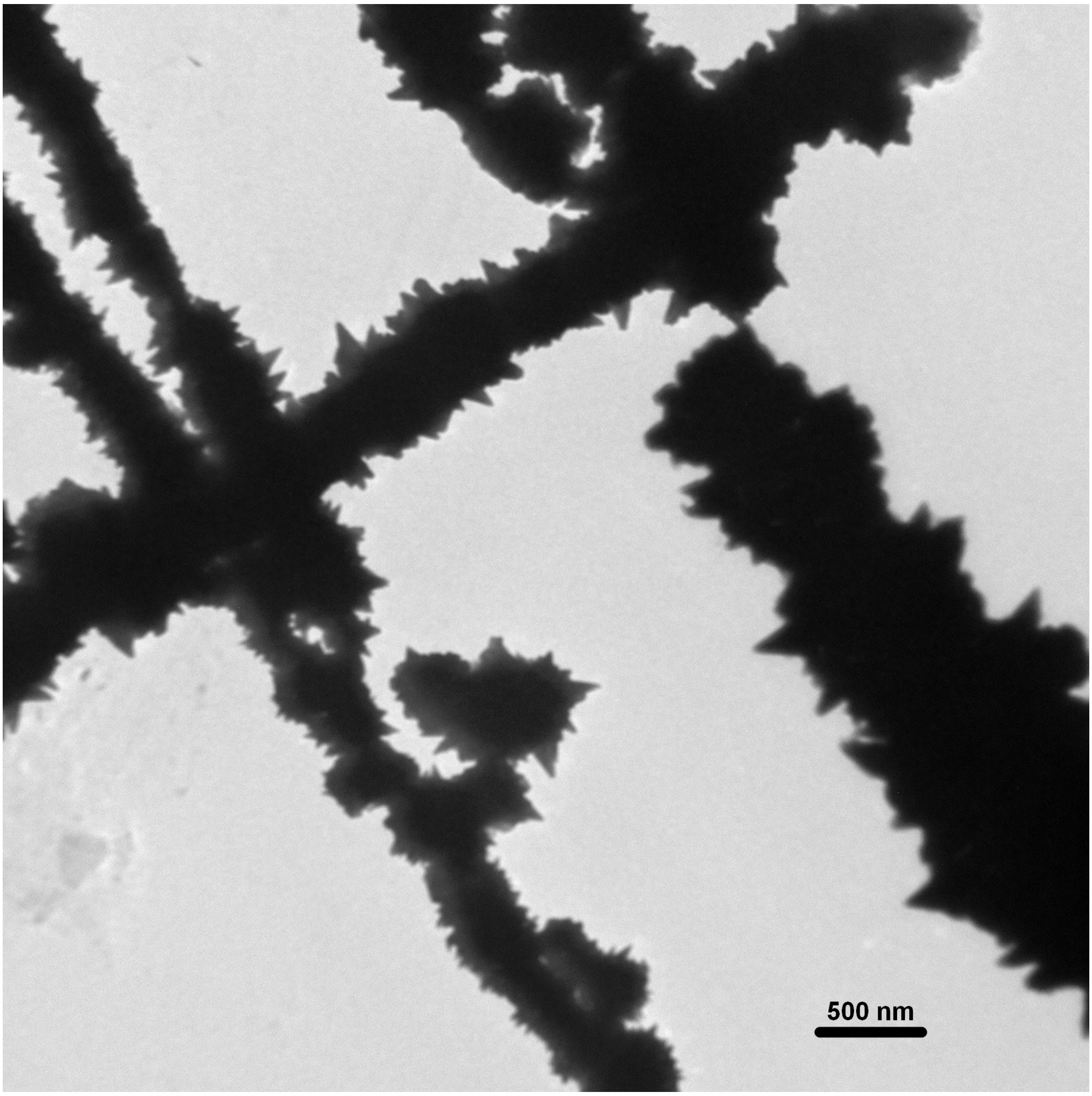

Figure S7. TEM image of ***NiFs***

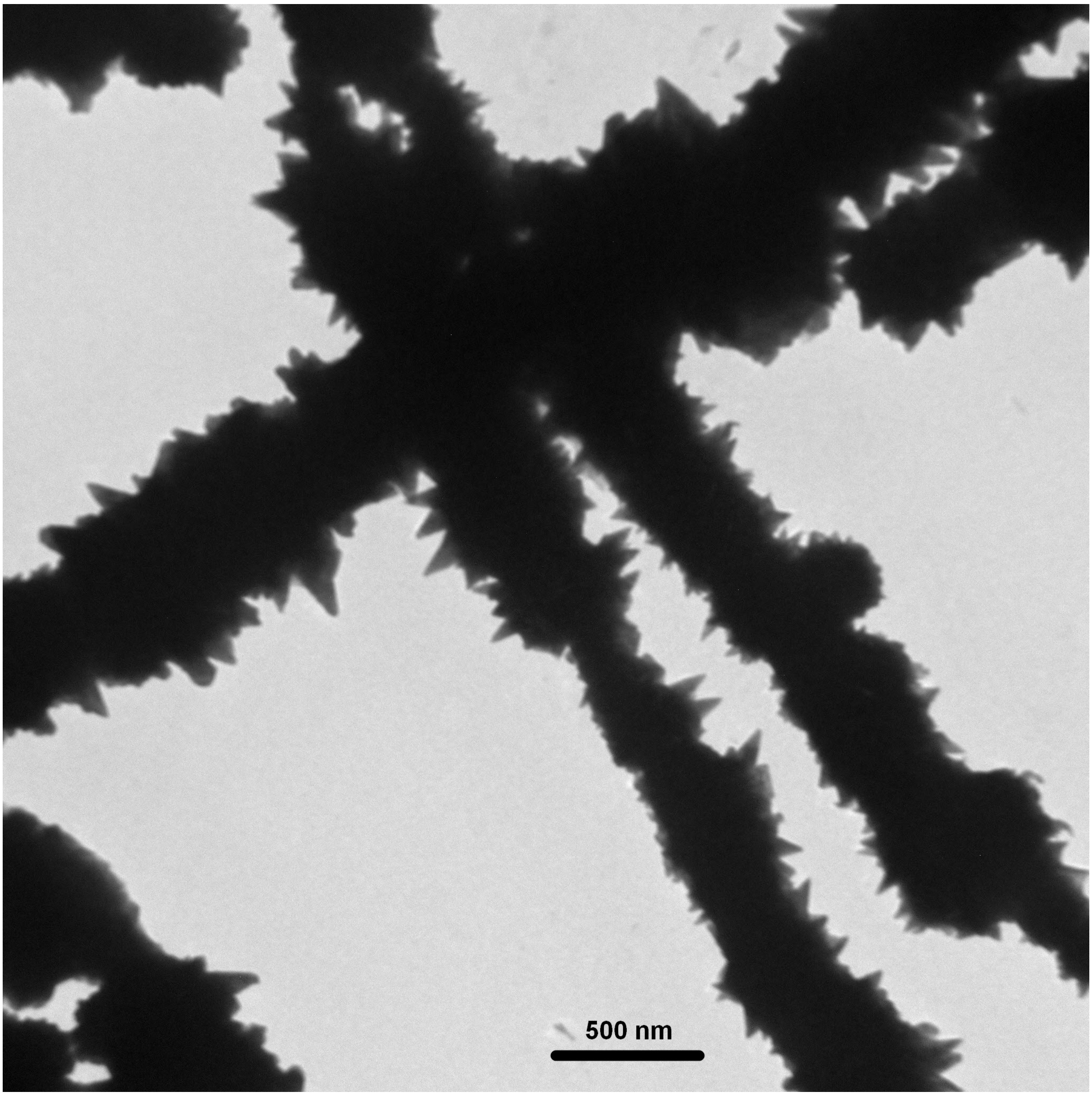

Figure S8. TEM image of ***NiFs***

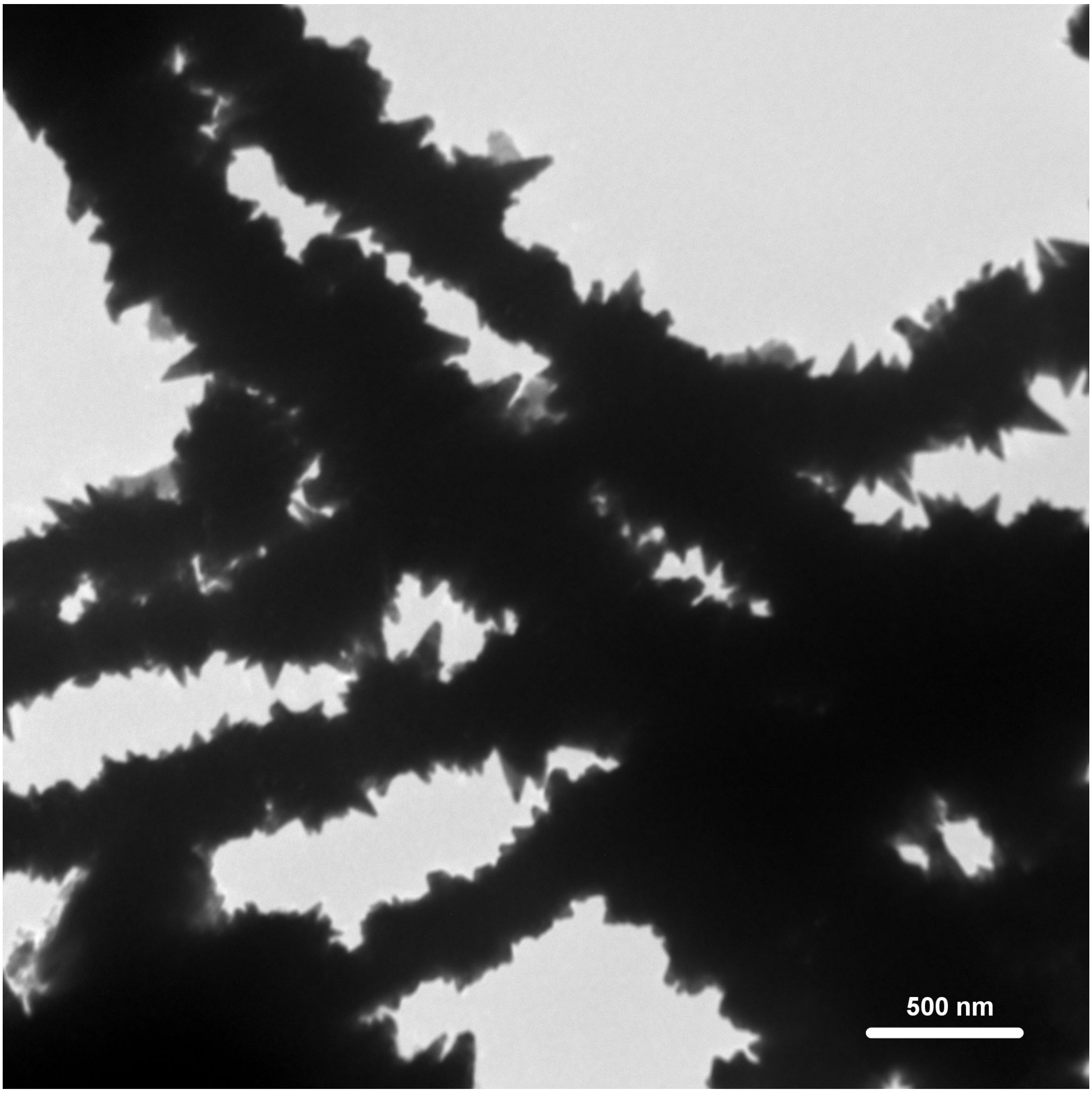

Figure S9. TEM image of ***NiFs***

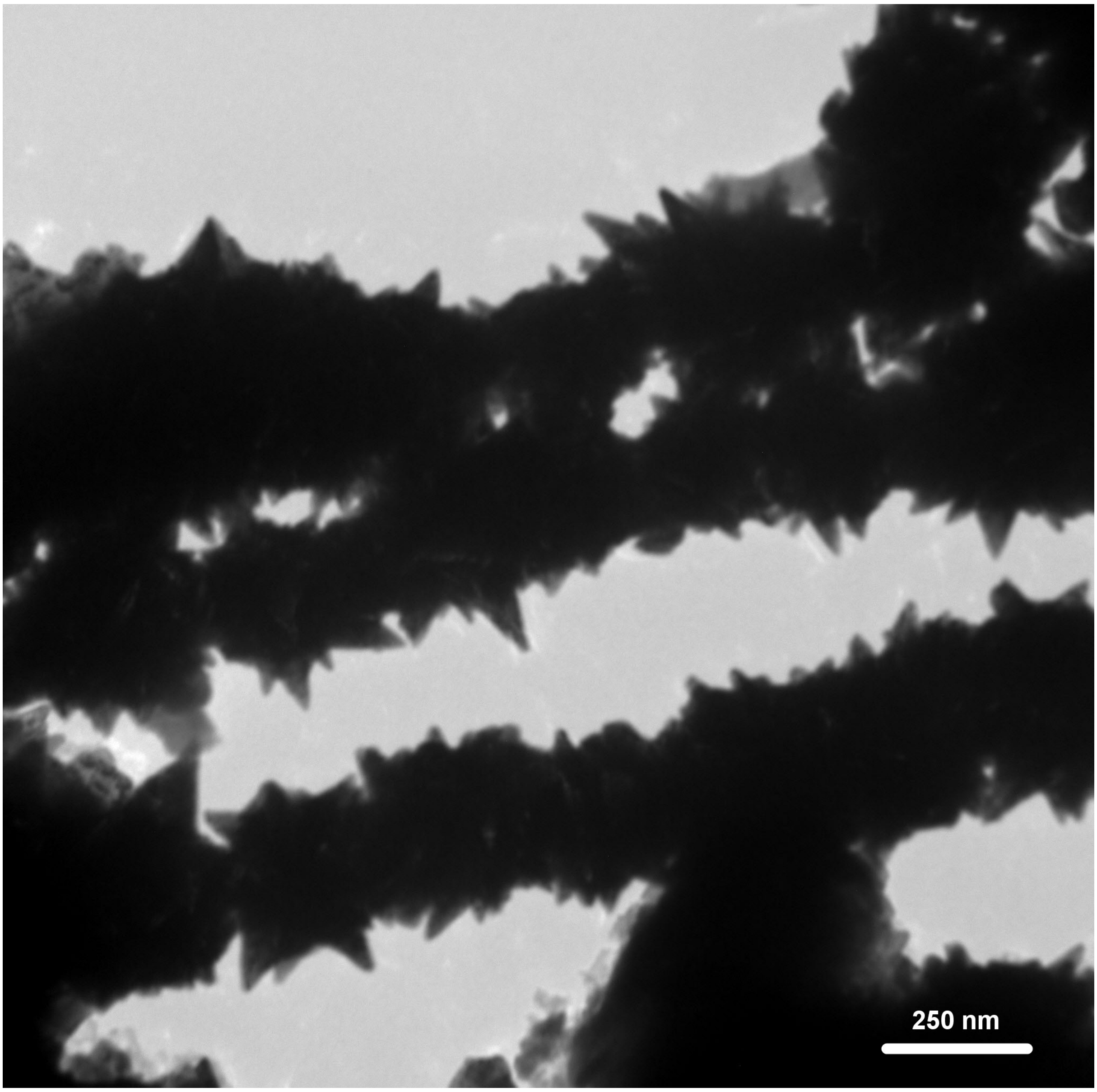

Figure S10. TEM image of ***NiFs***
